# Geographic Partitioning of Dengue Virus Transmission Risk in Florida

**DOI:** 10.3390/v13112232

**Published:** 2021-11-05

**Authors:** Caroline J. Stephenson, Heather Coatsworth, Christy M. Waits, Nicole M. Nazario-Maldonado, Derrick K. Mathias, Rhoel R. Dinglasan, John A. Lednicky

**Affiliations:** 1 Emerging Pathogens Institute, University of Florida, Gainesville, FL 32608, USA; c.stephenson@ufl.edu (C.J.S.); h.coatsworth@ufl.edu (H.C.); c.waits@ufl.edu (C.M.W.); nicole.nazarioma@ufl.edu (N.M.N.-M.); d.mathias@ufl.edu (D.K.M.); 2 Department of Environmental and Global Health, University of Florida, Gainesville, FL 32608, USA; 3 Department of Infectious Diseases and Immunology, University of Florida, Gainesville, FL 32608, USA; 4 Navy Entomology Center of Excellence, Naval Air Station, Jacksonville, FL 32212, USA; 5 Institute of Food and Agricultural Sciences, Florida Medical Entomology Laboratory, University of Florida, Vero Beach, FL 32962, USA

**Keywords:** *Aedes aegypti*, dengue virus, transmission potential, Florida

## Abstract

Dengue viruses (DENVs) cause the greatest public health burden globally among the arthropod-borne viruses. DENV transmission risk has also expanded from tropical to subtropical regions due to the increasing range of its principal mosquito vector, *Aedes aegypti*. Focal outbreaks of dengue fever (dengue) in the state of Florida (FL) in the USA have increased since 2009. However, little is known about the competence of *Ae. aegypti* populations across different regions of FL to transmit DENVs. To understand the effects of DENV genotype and serotype variations on vector susceptibility and transmission potential in FL, we orally infected a colony of *Ae. aegypti* (Orlando/ORL) with low passage or laboratory DENV-1 through -4. Low passage DENVs were more infectious to and had higher transmission potential by ORL mosquitoes. We used these same DENVs to examine natural *Ae. aegypti* populations to determine whether spatial distributions correlated with differential vector competence. Vector competence across all DENV serotypes was greater for mosquitoes from areas with the highest dengue incidence in south FL compared to north FL. Vector competence for low passage DENVs was significantly higher, revealing that transmission risk is influenced by virus/vector combinations. These data support a targeted mosquito-plus-pathogen screening approach to more accurately estimate DENV transmission risk.

## 1. Introduction

Dengue viruses (DENVs) belong to the genus *Flavivirus* in the family *Flaviviridae Ae.* There are four genetically related dengue viruses termed DENV-1, DENV-2, DENV-3, and DENV-4. These arthropod-borne viruses (arboviruses) are transmitted to humans through the bite of an infected *Aedes* species mosquito. The primary vector of these viruses is *Aedes aegypti* (*Ae. aegypti*). Dengue viruses are the causative agents of dengue fever (“dengue”) and a severe form of dengue termed dengue hemorrhagic fever. Dengue manifests a wide spectrum of clinical signs that range from subclinical (inapparent) disease to illnesses accompanied by fever, aches and pains (eye pain, and muscles, joint, and bone aches and pains), nausea, vomiting, and a rash [1]. Severe dengue can result in internal bleeding, shock, and death; bleeding can occur from the nose or gums, and blood can be present in vomit or stool. According to the World Health Organization, dengue is the most critical mosquito-borne disease in the world [1]. Between 1990 and 2013, symptomatic infections from DENV quadrupled, and nearly half of the world’s population is currently at-risk for DENV infection since the principal mosquito vector has extended its home range and now thrives in countries wherein it was previously absent [2,3,4]. Thus, DENVs have by now caused outbreaks of dengue in over 120 countries, with an estimated 400,000 infections and 10,000 deaths globally each year [1,4]. Despite a lengthy historical record of dengue outbreaks, there is still no effective treatment for this disease, and only one licensed vaccine is available. However, as some seronegative recipients have developed clinical signs reminiscent of antibody-dependent enhancement of DENV infection leading to severe dengue, this vaccine is limited to those with proof of previous dengue infection [5]. Due to the lack of preventative interventions, the current best practices for DENV prevention rely heavily on mosquito vector control, although this approach is insufficient to completely prevent DENV transmission [6,7,8,9,10,11].

**Figure 1:**
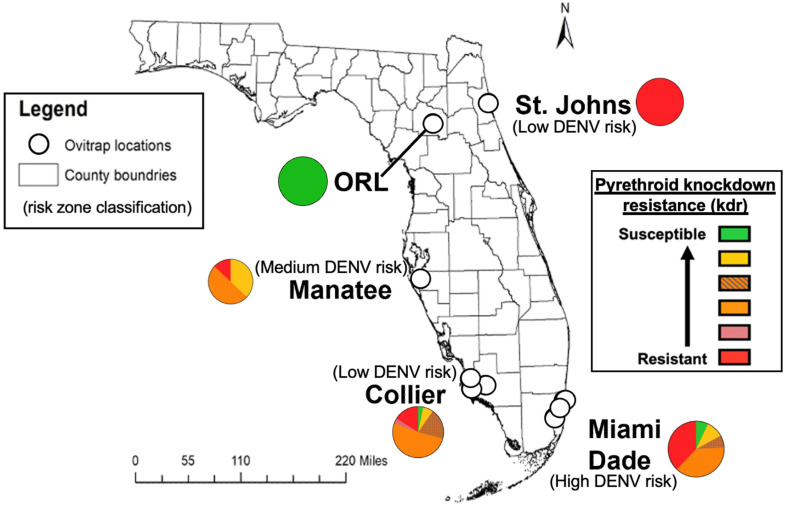
*Aedes aegypti* collection locations for four field-derived colonies from different historical DENV risk zones and one laboratory colony in Florida, USA., with reported *kdr* genotype frequency distributions for pyrethroid insecticide resistance shown as part-of-whole graphs.

Though *Ae. aegypti* are generally considered susceptible to infection with and capable of transmitting DENVs (i.e., competent vectors), field-caught and laboratory-bred *Ae. aegypti* exhibit considerable variation in vector competence for DENV. This variation is influenced by virus serotype and strain, as well as mosquito genetics, mosquito metagenome, and environmental factors [12,13,14,15,16,17,18,19,20,21,22]. As such, the overwhelming number of estimates of *Ae. aegypti* DENV competency derived from DENV-2 laboratory strain New Guinea C (NGC) [12] and of mosquito laboratory colony infections lack the granularity and biological complexity to inform the vector competency for other DENV serotypes and strains in other mosquito populations. This narrow insight limits our understanding of potential serotype- and strain-specific interactions that could influence real-world scenarios. As such, it is prudent to test the vector competence of local mosquitoes from a geographic region of interest to evaluate the focally relevant risk for DENV transmission. In Thailand, *Ae. aegypti* from areas with high human dengue incidence were shown to have higher vector competence than *Ae. aegypti* from regions of low dengue incidence [23]. However, in Cuba, *Ae. aegypti* sampled from areas categorized as low- and high-risk based on long-term trends in local human dengue case data demonstrated no significant differences in vector competence for a local circulating strain of DENV-1, with transmission rates ranging from 10–25% (Figure 1) [24]. Therefore, to understand the risk that dengue poses to a community, it is important to prioritize the comprehensive examination of vector competence of local vectors collected across spatially risk-partitioned zones.

Having previously broken the cycle of dengue from the United States (USA) through insecticide use and source reduction, the state of Florida (FL) is vulnerable to the establishment of all four serotypes of DENV in mosquito populations leading to subsequent local transmission in human populations [6]. *Aedes aegypti* mosquito populations have resurged following widespread displacement by *Ae. albopictus* in the mid-1990s and are now present throughout much of the state [25]. In addition, hundreds of travel-associated dengue cases are recorded each year in FL, and these correlate with sporadic local transmission, acting as seeding events [26]. Caribbean countries, particularly Haiti, are a key source of imported DENV in FL [27,28]. Nearly all local transmission of DENV in the state has occurred in its southern half, with the majority of cases in the southernmost counties (Co.) [26,29]. There were 28 locally acquired DENV-1 cases in Monroe Co. in 2009 and 65 cases in 2010, as well as 29 cases in Martin Co. in 2013 and 71 cases in Monroe and Miami-Dade Co.’s in 2020 [27,30,31,32,33]. To date, vector competence studies for DENV in FL have been completed for *Ae. aegypti* from two counties, Monroe (Key West) and Indian River (Vero Beach), and these studies used DENV-1 strains from either Key West or Puerto Rico (PR), or a DENV-2 strain from PR, respectively [15,29,34]. In spite of the sporadic local transmission of DENV in FL during the past twelve years [26], little proactive work has been done to quantify and characterize the vector competence of *Ae. aegypti* mosquito populations from different regions of the state.

It has become increasingly clear that insecticide resistance can influence vector competence. Pyrethroids are commonly used insecticides for controlling both nuisance mosquitoes and disease vectors globally and pyrethroid resistance in *Ae. aegypti* is a significant issue in FL [35]. Pyrethroid use is widespread globally, and insecticide resistance is prevalent but variable throughout the state of Florida [36]. There are two common nonsynonymous mutations to the voltage-gated sodium channel transmembrane protein gene in *Ae. aegypti* resulting from a 1016 valine to isoleucine (V1016I) and/or a 1534 phenylalanine to cysteine (F1534C) change; these mutations are known as knockdown resistance (*kdr*), and they reduce pyrethroid activity through target-site insensitivity. It was shown that a pyrethroid-resistant *Ae. aegypti* strain with *kdr* mutations selected from mosquitoes collected in Monroe Co., FL, had a higher DENV-1 dissemination rate compared to its unselected, susceptible counterpart [37]. A study of a laboratory colony of *Ae. aegypti* from Orlando, FL (ORL), with both pyrethroid-susceptible and resistant phenotypes, found that the pyrethroid-resistant mosquitoes had higher disseminated Zika virus titers [38]. Although the mechanism by which pyrethroid resistance-conferring loci influence vector competence is unclear, the pyrethroid resistance status of field-derived mosquitoes should also be considered in estimating vector competence and arbovirus transmission risk.

Herein, we examined two facets of vector competence, susceptibility (midgut infection) and transmission potential (virus detection in saliva), to understand if *Ae. aegypti* from various DENV risk zones in FL have varying levels of competence. Our defined DENV risk zones are based on historical DENV cases/detections, where no local DENV cases or DENV-positive mosquitoes have been reported, north FL (low risk), where no local DENV cases but DENV-positive mosquitoes have been detected, central FL (medium risk), and numerous local DENV cases and DENV-positive mosquitoes have been reported, south FL (high risk) [28,30,33,39]. We assessed competency across DENV 1, 2, 3, and 4, using four laboratory (L) stock viruses (DENV-1 L, DENV-2 L, DENV-3 L, DENV-4 L), and two low-passage field isolates from human specimens from Haiti ((H): DENV-1 H and DENV-4 H). We also measured and compared infection rates and virus titers in mosquito tissues (infection intensities) between pyrethroid-susceptible ORL mosquitoes and *Ae. aegypti* populations from the aforementioned risk zones. These data were used to understand how intra- and inter-serotype variation may affect vector competence and, consequently, DENV transmission risk in the state.

## 2. Materials and Methods

### 2.1. Mammalian Cell Culture and Virus Propagation

Mammalian cell culture and virus propagation methods for this experimental design were conducted similarly to previously published methods [17]. Briefly, for the preparation of virus stocks, Vero E6 cells grown as monolayers were seeded onto the 75 cm^2^ growing surfaces of filter-cap T75 cell culture flasks and incubated until they attained 80% confluency. The cells were then individually inoculated with DENV-1 through DENV-4 laboratory stock viruses (DENV-1 L (VR-1856™), DENV-2 L (VR-1584™), DENV-3 L (VR-1256_FD™), DENV-4 L (VR-1490™)) obtained from the American Type Culture Collection (ATCC.org) as well as a low passage (second passage) DENV-1 (DENV-1 H) isolate from a child in Haiti (strain: Haiti/1207/2014: KT279761.2) and a low passage (second passage) DENV-4 isolate, also from a child in Haiti (strain: Haiti/0075/2015: MK514144.1) (Table A1). All virus stocks were collected between 7- and 9-days post-inoculation when approximately 50% of the cells showed virus-specific cytopathic effects (CPE) and were tittered, stabilized with 10% *w*/*v* molecular grade trehalose and cryopreserved as previously described [17]. All virus titers and infectious doses for these experiments are listed in Table A1.

### 2.2. Risk Zone Classification

We chose to test *Ae. aegypti* from four counties to represent current day low-, medium-, or high-risk areas for dengue virus transmission in FL. St. Johns Co. in north FL (Figure 1) was classified as “low risk” because no dengue-positive mosquito pools nor local human cases have been reported from this county since dengue cases returned in FL in 2009 after over fifty years without local cases [39]. Manatee Co. in southwest FL was classified as “medium risk” because there was a report of dengue-positive *Ae. aegypti* (DENV-4) in 2016 and 2017, but no previous local dengue transmission has been detected since 2009 [40]. Miami-Dade Co. in south FL was classified as “high risk” because there have been reports of both dengue-positive mosquito pools (unpublished) and human dengue cases in multiple years since 2009 [30,33]. Collier Co. was added as an additional “low risk” county (with no dengue-positive mosquito pools nor human cases) to act as a “low risk” south FL comparator for Miami-Dade Co., since both counties are further south in the sub-tropical zone, and reflect climates with warmer temperatures and higher relative humidity on average than St. Johns Co. in northeastern FL [41,42].

### 2.3. Field-Caught Ae. aegypti

We obtained field-caught G_0_ *Ae. aegypti* eggs from Anastasia Mosquito Control District in St. Johns Co., Collier Mosquito Control District (Collier Co., Naples, FL, USA), and Miami-Dade Mosquito Control District (Miami-Dade Co., Miami, FL, USA). We also received Manatee Co., Bradenton, FL, USA *Ae. aegypti* eggs (F_7_) from Dr. Derrick Mathias at the Florida Medical Entomology Laboratory at the University of Florida. Upon receipt, the eggs were hatched, and adult mosquitoes were cold anesthetized and morphologically verified as *Ae. aegypti* by expert identification. F_2_ generation *Ae. aegypti* from Miami-Dade (MD) and St. Johns Co’s (SJ) were used for the following studies. The F_7_ generation of *Ae. aegypti* from Manatee Co. (MAN) was used. The Collier Co. (COL, Gainesville, FL, USA) comparator group was obtained from the field in 2018 and was reared in the laboratory from 2018 (exact generation unknown). The field colonies (homogenized pooled legs from the F_1_ generation of MD and SJ, F_7_ generation of MAN, and unknown generation of COL) were screened via RT-PCR for the presence of Cell Fusing Agent virus (CFAV) (Table A2), a potentially confounding insect-specific virus (ISV); all mosquito pools tested negative.

*Aedes aegypti* Orlando strain (ORL) was obtained as adults from the United States Department of Agriculture’s Center for Medical, Agricultural and Veterinary Entomology (USDA-CMAVE) in Gainesville, Florida, and then established as a colony in our laboratory. Mosquitoes were reared in growth chambers set to 28 °C with 80% relative humidity and a neutral photoperiod regimen (12 h light/12 h dark).

### 2.4. Mosquito Rearing, Infection, and Tissue/Specimen Preparations

Four to seven days post-eclosion, female mosquitoes were separated and used in per os DENV infections. These females were artificially fed with a 2:2:1 human hematocrit type O+ blood (hematocrit) (Lifesouth Community Blood Centers, Gainesville, FL, USA): virus stock: heat-inactivated human serum (HIHS). After one hour, blood-engorged mosquitoes were kept for further analysis, and all other mosquitoes were discarded. We maintained the mosquitoes for 14 days on a 10% sucrose solution. On day 14, we cold anesthetized the mosquitoes, removed their legs and wings, and manually inserted their proboscis into a capillary tube (Drummond, Broomall, PA, USA) filled with 3 µL of 1:1 hematocrit: HIHS [17]. Each proboscis was withdrawn after 45 min or after 2 µL of hematocrit was imbibed. The remaining contents of the capillary tube were expelled into microcentrifuge tubes. Next, the midgut of each mosquito was dissected and individually stored in microcentrifuge tubes in DMEM, and we recorded which mosquitoes had blood in their bodies from the salivation assay. These experiments were repeated in triplicate for each of the six virus groups across all four field-caught mosquito strains and the ORL laboratory strain.

### 2.5. Mosquito Midgut and Saliva Analysis

Baby hamster kidney fibroblast cells (BHK-21, ATCC^®^ CCL-10™) (a generous gift from the Dimopoulos laboratory at Johns Hopkins University) were seeded onto 24-well plates at a density of 5 × 10^4^/well and incubated at 37 °C with 5% CO_2_ until nearly confluent. Each mosquito midgut was homogenized by using a Bullet Blender (Nextadvance, NY, USA), adjusted to speed setting 8 for 3 min, and serially diluted 10-fold before adding 100 µL of each dilution series to the 24-well plate. We used 0.8% *w*/*v* methylcellulose media for our overlay and incubated the plates for six days (except for DENV-4 H and L that were incubated for 5 days due to the quicker development of plaques). The plates were fixed in a 1:1 methanol/acetone solution with 1% *w*/*v* crystal violet for an hour, after which plaques were counted and virus titer expressed as plaque-forming units per mL (PFU/mL).

The DENV-1 laboratory strain (DENV-1 L) did not clearly form plaques on BHK-21 or Vero E6 cells after numerous attempts. Cytopathic effects could be visualized under a phase contrast inverted microscope when the virus was inoculated onto Vero E6 cells. Therefore, we seeded 48-well plates with Vero E6 cells at a density of 3 × 10^4^/well, and upon reaching 70% cell confluency, we individually inoculated each well with 50 µL of midgut homogenate from ORL mosquitoes exposed to DENV-1 L. The cultures were monitored for a total of 27 days wherein media was replenished and inoculum re-passaged as needed or when cells became overly confluent; this was done to ensure that CPE could be detected even if the starting virus concentration was low. The readout for positive midgut samples was a dichotomous qualitative outcome of “infected” or “not infected” based on the presence or absence of CPE compared to the mock-inoculated (with DMEM) wells grown in parallel conditions throughout the twenty-seven days.

Ribonucleic acid (RNA) was purified from saliva samples from ORL mosquitoes that had a DENV-positive midgut, either by plaque assay (DENV-1 H, DENV-2 L, DENV-3 L, DENV-4 H, and DENV-4 L) or by cell culture (DENV-1 L) using a QIAamp Viral RNA Mini Kit (Qiagen, Valencia, CA, USA). Each RNA sample was subjected to rtRT-qPCR using a dual-target pan-DENV system (Table A2) (Superscript III, Waltham, MA, USA) and run as technical duplicates on a BioRad CFX96 Touch Real-Time PCR Detection System. A standard curve of each virus was run for each assay to determine the virus-specific regression analysis for estimating PFUe/mL.

### 2.6. Statistical Analyses

All statistical analyses were performed with SPSS statistical software (IBM Corp. Released 2020. IBM SPSS Statistics for Windows, Version 27.0. Armonk, NY, USA: IBM Corp, accessed 2 September 2021). Figures were created using GraphPad Prism Version 9.1.1 (223), for Mac, GraphPad Software, San Diego, CA, USA, www.graphpad.com (accessed on 2 September 2021) or Microsoft Powerpoint Version 16.53 for Mac. Data for ORL and COL exposed to DENV-4 H and DENV-4 L were reanalyzed from Stephenson et al., 2021 to provide a complete analysis and comparison between all virus groups and mosquito populations [17]. In all tables and figures, numbers/bars/scatter plots bearing at least one of the same alphabetical letters are statistically similar in their values, whereas numbers/bars/scatter plots that do not have an alphabetical letter in common are statistically different. For example, COL had a statistically significant higher transmission potential (11%) for DENV-4 H compared to MD (2%) but was not statistically higher than MAN (4%). Therefore, 11% is denoted with an “A”, 2% is denoted with a “B”, but 4% is denoted with “AB” because this rate was not statistically different from either 11% or 2%.

To examine infection rates, a multilevel logistic regression was used with one fixed effect predictor; the binary response variable, midgut infection status, “non-infected = 0” or “infected = 1”, and the fixed variable included each strain of mosquitoes exposed to one of the six virus groups (e.g., St. Johns *Ae. aegypti* exposed to DENV-1 H). Sample sizes are listed in Table A3. We evaluated the potential for “batch effect” by including experimental replicate number as a random effect variable. The random effect predictor was significant (*p* = 0.027) (as such, it was incorporated as a factor in the full model). We report odds ratios (ORs) and *p*-values for each pairwise combination of IRs. Significant statistical significance (α = 0.05) is denoted via groups assigned different letters.

The transmission potentials (TPs) of field mosquitoes were analyzed via binary logistic regression, with sample sizes listed in Table A3. The binary saliva infection status was “non-infected = 0” or “infected = 1”. There was no significant batch effect for saliva samples, so we moved forward with a univariate analysis of saliva-positivity rates from field mosquitoes exposed to one of six different DENVs. We reported ORs and *p*-values for each pairwise combination of TPs. Statistical significance (α = 0.05) is denoted via groups assigned different letters.

Average titers in log_10_ PFU/mL (transformed for normality) were compared using a mixed-methods ANOVA between each virus group and field mosquito population for both midgut and saliva samples. We included batch effect as the random effect variable to control for replicate effects. The fixed effect was a combination of virus strain and mosquito strain. Both midgut and saliva residuals for log_10_ titer passed the Shapiro–Wilk test for normality, and the outcome variable passed Levene’s test for equality of variances.

### 2.7. kdr Genotyping

A previously published qPCR assay for the detection of two *kdr* single nucleotide polymorphisms (SNPs) coding for amino acids located at positions 1016 and 1534 of the *NA_V_* gene was adapted for this work (Table A3) [36,43,44]. Forty eggs from each field mosquito colony were homogenized (COL (generation unknown), MAN (F_8_), MD (F_3_), and SJ (F_3_)). Fifteen individual mosquito midgut samples (COL and MD) or 15 individual whole bodies (MAN and SJ) were also homogenized to compensate for the desiccation of some eggs that likely resulted in no signal for the assay. Final sample sizes were *n* = 31 for COL, *n* = 30 for MAN, *n* = 29 for MD, and *n* = 31 for SJ. ORL was used as the pyrethroid-susceptible control and Puerto Rico (PR) *Ae. aegypti* (a kind gift from the USDA-CMAVE), as the pyrethroid-resistant control for this assay. We also included a heterozygote control that contained one ORL and one PR mosquito. The Applied Biosystems™ SYBR™ Select Master Mix for CFX was used with cycling conditions of 50 °C for 2 min, 95 °C for 2 min, and 45 cycles of 95 °C for 15 s and 60 °C for thirty seconds. There was a final melt curve with a ramp from 60 °C to 95 °C with reads every 0.3 °C, and genotypes were determined by melt curve analysis. For the 1016 SNP, amplicons from a pyrethroid-susceptible mosquito had a melting temperature of 85.6 °C, amplicons from resistant mosquitoes had a melting temperature of 77 °C, and heterozygotes had a peak at both temperatures. For the 1534 SNP assay, amplicons from a susceptible mosquito had a melting temperature of 79.3 °C, and amplicons from a resistant mosquito had a melting temperature of 84.4 °C. Heterozygotes had two peaks at both previously listed temperatures. We used genotype information from each mosquito per population to understand the distribution of genotype frequencies across each colony and visualized these results using pie charts.

## 3. Results

### 3.1. Serotype-Specific Vector Competence of the Ae. aegypti (ORL) Colony

**Figure 2:**
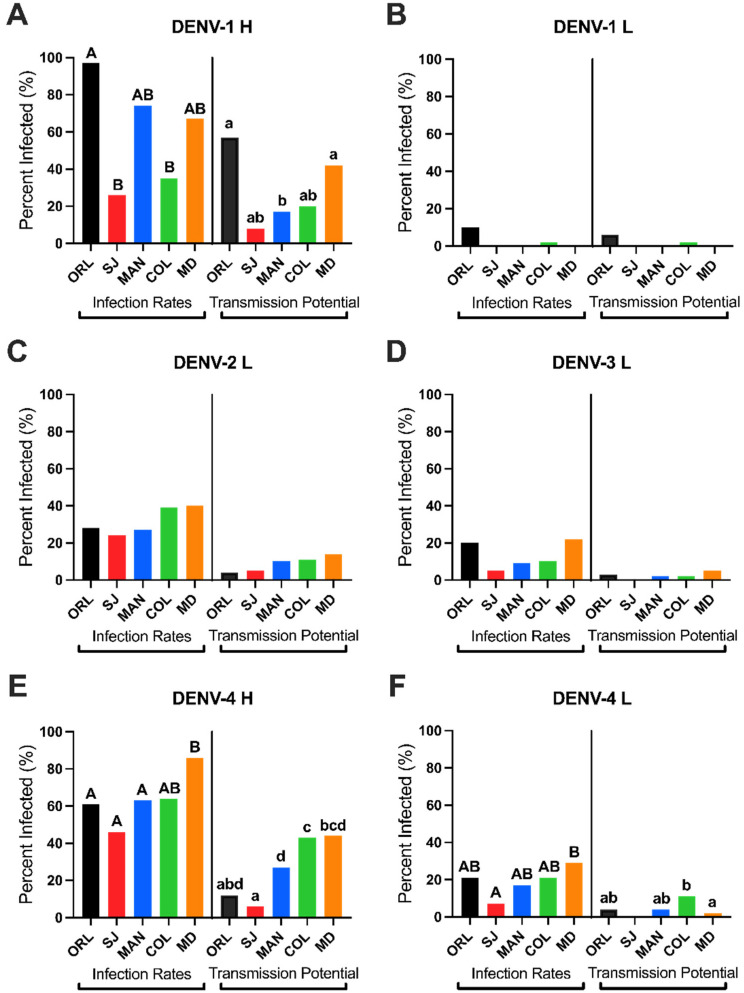
Infection rates and transmission potentials of Floridian *Aedes aegypti* mosquitoes (exposed to (**A**) DENV-1 Haiti, (**B**) DENV-1 laboratory, (**C**) DENV-2 laboratory, (**D**) DENV-3 laboratory, (**E**) DENV-4 Haiti, (**F**) DENV-4 laboratory) were highest for south FL (COL and MD) and laboratory (ORL) populations with low passage virus strains (DENV-1 H and DENV-4 H). Differences in alphabetical capital letters (A or B) denote statistical significance between infection rates within each virus group. Differences in lowercase letters (a, b, c, or d) denote statistical significance between transmission potentials within each virus group. Bars are averaged from three experimental replicates. Groups without any statistically significant differences do not have any alphabetical letter labeling. Significance is measured using an alpha level of 0.05 via multilevel logistic regression (infection rates) or binary logistic regression (transmission potentials).

We developed a vector competence baseline for *Ae. aegypti* ORL mosquitoes to refine the community’s perception of the utility of this colony in such studies. The ORL colony used herein has been continuously reared at the USDA (Gainesville, FL) since 1952, is pyrethroid susceptible, and is not infected with cell-fusing agent virus (CFAV), an insect-specific virus that has been shown to influence arbovirus infections of mosquito cells [22]. We performed pairwise comparisons of midgut infection rates (IRs) to evaluate susceptibility to infection (Figure 2 and Table 1). Figure 2 displays the trends in infection rates and transmission potentials across each mosquito population by virus group. Table 1 displays all raw infection rates and transmission potentials with statistical comparisons across virus groups and across virus groups. IRs represent the average number of DENV-infected midguts out of the total number of exposed mosquitoes per group across three experimental replicates. In pairwise comparisons of DENV-exposed ORL mosquitoes, midgut IR was highest for DENV-1 H with 97%, followed by DENV-4 H (61%), DENV-2 L (28%), DENV-4 L (21%), DENV-3 L (20%), and DENV-1 L (10%). The odds of midgut infection were significantly higher for ORL mosquitoes exposed to DENV-1 H compared to DENV-1 L (Odds ratio (OR): 219.9, *p*-value (*p*) < 0.001), DENV-2 L (OR: 56.2, *p* < 0.001), DENV-3 L (OR: 120.4, *p* < 0.001), DENV-4 H (OR: 14.5, *p* < 0.001), and DENV-4 L (OR: 79.5, *p* < 0.001). There were also higher odds of midgut infection for DENV-4 H compared to DENV-1 L (OR: 15.1, *p* < 0.001), DENV-2 L (OR: 3.9, *p* = 0.001), DENV-3 L (OR: 8.3, *p* < 0.001), and DENV-4 L (OR: 5.5, *p* < 0.001). Lastly, DENV-2 L had higher odds of midgut infection than DENV-1 L (OR: 3.8, *p* = 0.012).

### 3.2. Low Passage DENV Infections Have Elevated Midgut and Saliva Infection Intensities in Ae. aegypti ORL Mosquitoes

**Figure 3:**
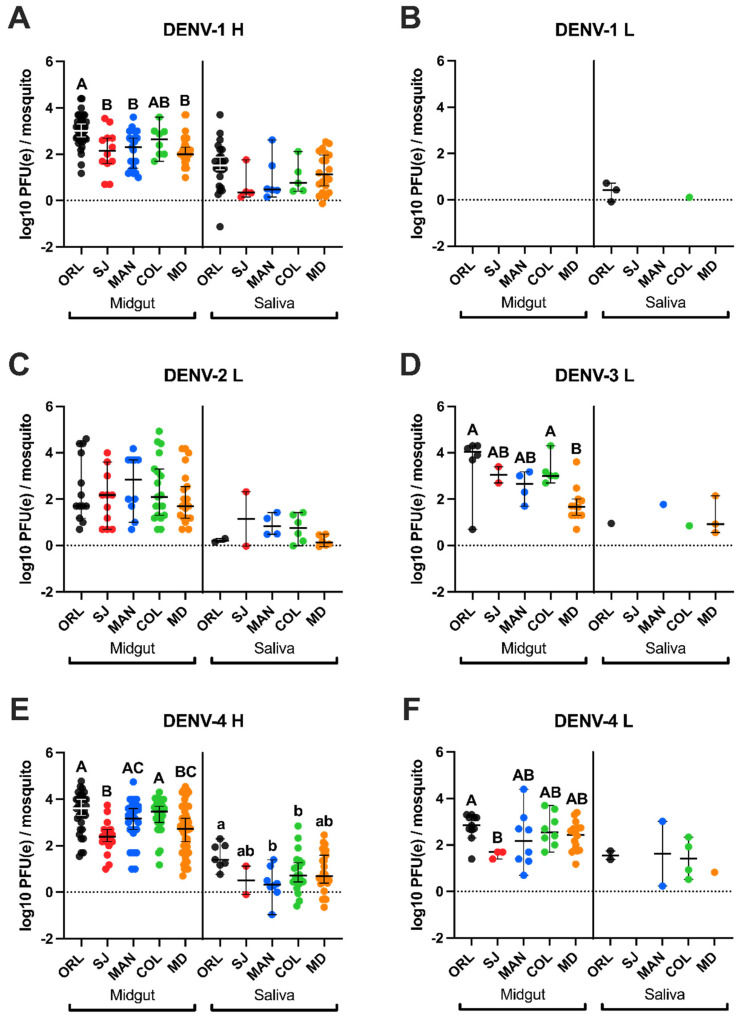
Infection intensities for Floridian *Aedes aegypti* (exposed to (**A**) DENV-1 Haiti, (**B**) DENV-1 laboratory, (**C**) DENV-2 laboratory, (**D**) DENV-3 laboratory, (**E**) DENV-4 Haiti, (**F**) DENV-4 laboratory) were elevated for low passage DENV strains (DENV-1 H and DENV-4 H) compared to laboratory virus strains. Differences in alphabetical capital letters (A, B, C) denote statistical significance between midgut infection intensities within each virus group. Differences in lowercase letters (a or b) denote statistical significance between saliva infection intensities within each virus group. Groups without any statistically significant differences do not have any alphabetical letter labeling. Significance is measured using an alpha level of 0.05 via mixed methods ANOVA.

We compared infection intensities among ORL infected with DENVs and saw higher titers for low passage viruses (Figure 3 and Table 2). We observed higher mean midgut titers for mosquitoes infected with DENV-1 H compared to DENV-2 L (*p* = 0.044). The average titer for midgut tissues infected with DENV-4 H was significantly higher than titers for DENV-1 H (*p* = 0.049), DENV-2 L (*p* < 0.001), and DENV-4 L (*p* = 0.038). The average saliva infection intensity of ORL for DENV-1 H was significantly higher than DENV-1 L (*p* = 0.024) and DENV-2 L (*p* = 0.035). Similarly, the saliva infection intensity for DENV-4 H was significantly higher than DENV-1 L and DENV-2 L (*p* = 0.034 and 0.042).

### 3.3. Field-Derived Ae. aegypti from South FL Have Comparatively Greater Vector Competence

We compared IRs for each DENV risk group: *Ae.* *aegypti* from “low risk” St. Johns Co. (SJ) in north FL, “low risk” Collier Co. (COL) in south FL, “medium risk” Manatee Co. (MAN), and “high risk” Miami-Dade Co. (MD). There were significantly higher odds of midgut infection for MD exposed to DENV-4 H compared to both MAN (OR: 3.0, *p* = 0.024) and SJ (OR: 6.4, *p* < 0.001). There were also significantly higher odds (OR: 2.8, *p* = 0.035) of midgut infection for MD *Ae.* *aegypti* exposed to DENV-4 L compared to SJ. When comparing the two south FL mosquito groups, COL and MD had similar midgut IRs across all virus pairings.

Transmission potential was higher for MD *Ae.* *aegypti* exposed to DENV-1 H (42%) compared to MAN (17%) (OR: 4.8, *p* = 0.012). There was also significantly higher DENV-4 H transmission potential for both MD (44%) and MAN (27%) compared to SJ (6%). Miami-Dade Co. (MD) was 8.2 times more likely to have DENV-4 H detected in their saliva compared to SJ, and MAN was 5.9 times more likely to have DENV-4 H detected in their saliva compared to SJ. There were no significant differences among the TPs of laboratory viruses between SJ, MAN, or MD. When comparing the two south FL mosquito groups, COL had higher DENV-4 L transmission potential (OR: 15.0, *p* = 0.03) compared to MD, but the remaining comparisons were similar.

### 3.4. Higher Infection and Transmission Potentials for Low Passage DENVs in Floridian Field Ae. aegypti

Overall, we found higher IRs and TPs for the low passage viruses compared to laboratory viruses. For example, COL had higher odds of DENV-4 H midgut infection compared to every other virus group for COL (DENV-1 H, OR: 6.7, *p* < 0.001; DENV-1 L, OR: 80.3, *p* < 0.001; DENV-2 L, OR: 3.7, *p* = 0.008; DENV-3 L, OR: 23.4, *p* < 0.001; DENV-4 L, OR: 9.4, *p* < 0.001). Miami-Dade Co. (MD) had a significantly higher odds of DENV-1 H transmission potential compared to DENV-2 L (OR: 4.8, *p* = 0.012), DENV-3 L (OR: 5.07, *p* = 0.038), and DENV-4 L (OR: 32.3, *p* < 0.001).

### 3.5. Low Passage DENV-4 Attained Highest Titers in Field Ae. aegypti Midgut Samples but Not the Highest Genome Copies in Saliva

There were no significant differences in infection intensities for SJ *Ae. aegypti*. MAN had significantly higher average DENV-4 H midgut titers, compared to DENV-1 H (*p* = 0.002) and DENV-4 L (*p* = 0.034), but DENV-4 L had higher average genome copies in saliva compared to DENV-4 H (*p* = 0.05). Miami-Dade Co. (MD) had the highest average DENV-4 H midgut titers in comparison to DENV-1 H (*p* = 0.008), DENV-2 L (*p* = 0.008), and DENV-3 L (*p* < 0.001). Collier Co. (COL) midgut infection intensity was higher for DENV-4 H than both DENV-1 H (*p* = 0.045) and DENV-2 L (*p* < 0.001). Even with higher DENV-4 H midgut titers observed across most of the mosquito populations, DENV-4 H saliva titers were comparable or lower than other virus groups for field *Ae. aegypti*.

### 3.6. Higher Infection Intensity for Laboratory Ae. aegypti (Orlando) Compared to Floridian Field Ae. aegypti

Orlando *Ae. aegypti* reached the highest average midgut titers compared to COL, MAN, MD, and SJ, except for DENV-2 L titers for MAN. Orlando (ORL) had significantly higher midgut titers for DENV-1 H compared to SJ (*p* = 0.002), MD (*p* < 0.001), and MAN (*p* < 0.001). Additionally, ORL had significantly higher midgut titers for DENV-3 L compared to MD (*p* < 0.001), and for DENV-4 H compared to SJ (*p* < 0.001) and MD (*p* < 0.001). Orlando (ORL) also had higher DENV-4 L midgut titers compared to SJ (*p* = 0.039). Finally, ORL had higher DENV-4 H titers in saliva compared to COL (*p* = 0.05) and MAN (*p* = 0.005).

### 3.7. Knockdown Resistance Allele Profiles in Field-Derived Ae. aegypti Inversely Trends with Vector Competence for DENV

The frequency distributions of *kdr* alleles are presented in Figure 1 and Table A4. St. John’s Co. (SJ) had the highest resistance profile, with 100% of tested mosquitoes bearing both resistance alleles (IICC). Miami-Dade Co. (MD) had the second-highest percentage of resistant mosquitoes (38% IICC) but had wide variability across *kdr* genotypes and did not have any fully pyrethroid susceptible mosquitoes. Collier Co. (COL) had 16% IICC mosquitoes, and 3.2% of mosquitoes carried the second most resistant genotype (IIFC). Collier Co. (COL) had the most variability in genotypes of the four groups tested and had a similar distribution to MD. Lastly, MAN had 13% IICC mosquitoes, 50% VICC (heterozygote for the 1016 allele and homozygous resistant for the 1534 allele), and 37% VVCC (homozygous susceptible for the 1016 allele and homozygous resistant for the 1534 allele). The field mosquito populations with higher pyrethroid resistance, based on their *kdr* genotypes, had the lowest measured vector competence. Orlando (ORL) is 100% susceptible (VVFF) at both loci and had the highest vector competence measures, followed by MD with 38% of mosquitoes bearing resistance genotypes. However, the SJ population had 100% resistance at these loci and was found to have the lowest competence for DENVs.

## 4. Discussion

We provided evidence that field *Ae. aegypti* populations from different DENV risk zones in FL vary in their vector competence profiles between the four DENV serotypes, and between low passage and laboratory virus strains. We observed a gradient of increasing competence from “low risk” SJ through “high risk” MD. Overall, MD had the highest IRs, second only overall to the ORL colony, and the highest susceptibility and transmission potential for both H strains compared to the laboratory viruses. In general, the highest IRs, TPs, and midgut titers occurred with either of the low passage viruses (DENV-1 H and DENV-4 H) compared to the laboratory viruses (DENV-1 L, DENV-2 L, DENV-3 L, and DENV-4 L), even though all, except DENV-1 L (10^5^ PFU/mL), had comparable blood meal titers (10^6^ PFU/mL). The DENV-1 L strain would not propagate to higher titers than 10^5^ PFU/mL regardless of cell line or duration of culturing in our hands. It is known that infectious dose is a contributing factor to the establishment of a midgut infection [45], but it is evident that the differences we identified in susceptibility occurred regardless of infectious dose standardization.

The four DENV laboratory stocks used in this study were previously found to share the same putative receptors in *Ae. aegypti* midguts [46]. Little is currently known about whether polymorphisms in these midgut receptors and associated proteins influence epithelial cell infections for field and laboratory DENVs. In humans, there is evidence of altered susceptibility of cell types by DENV-2 strains for both laboratory and field isolates [47]. In mosquitoes, similar DENV-2 midgut binding affinity was observed across various field isolates that had altered vector competence outcomes in *Ae. aegypti* [48]. Mutations that arise in arbovirus genomes can enhance virus dissemination and vector competence [49,50]. Previous work identified several DENV-2 mutants that bear amino acid changes that enhance virus dissemination out of the midgut [49]. Genetic differences among our virus strains, particularly in the envelope gene, could alter their midgut binding and cell tropism. At the nucleotide and amino acid level, DENV-2 and DENV-4 are the most divergent among the four serotypes, with DENV-1 and DENV-3 being most similar [51,52,53]. In particular, DENV-4 H and DENV-4 L share 93% nucleotide identity and 98% amino acid identity, but the DENV-4 L sequence has an additional 15 nucleotides in its 3′ UTR not seen in contemporary strains within genotype IIb [40]. We suspect that certain genetic changes arising from the continuous cell culture of the DENV laboratory stocks could be responsible for their lower fitness in *Ae. aegypti*. Now that serotype and strain-specific trends in vector competence have been established for both laboratory and field *Ae. aegypti*, as well as across laboratory and field strains of DENV, future work is needed to uncover the driving factors responsible for the phenotypic differences.

Several studies previously classified ORL mosquitoes as “refractory” compared to the Rockefeller strain of *Ae. aegypti*, but we suspect that this misclassification is likely a result of suboptimal vector-virus combinations that were used, especially since a lab-maintained, historical DENV-2 NGC strain was primarily tested [54,55]. An additional factor at play is that colonies referred to as “ORL” in the literature but from different labs have had different evolutionary histories post sub-colonization from the original ORL colony, which undoubtedly influences the degree of genetic similarity among colonies. In our study, ORL generally had higher susceptibility and competence measures, particularly for the low passage DENV strains, compared to field *Ae. aegypti*. The highest DENV titers were reported from ORL midgut samples compared to every other mosquito strain, except for DENV-2 titers for MAN. Additionally, DENV titers from ORL midgut and saliva samples were higher than several other mosquito strains for the various virus combinations. In the published literature, ORL has been reported to have a median DENV-2 midgut PFU of zero [56] and has also been found to have a DENV-2 midgut prevalence of zero in comparison to the laboratory colony known as Rockefeller [55]. Those studies had comparable or higher DENV-2 infectious doses (10^6^ or 10^7^ PFU) to our study, yet we measured an average and median DENV-2 concentration around 10^2^ PFU/midgut with an infection rate of 28% and transmission potential of 4%. Due to higher DENV competence of ORL, compared to FL field *Ae. aegypti*, we conclude that it would not serve well as a colony to use to ascertain risk of DENV transmission in a real-world setting but that it could serve as a useful model to study in vivo vector-virus interactions when high infection rates of low passage virus are needed. It appears that ORL has been previously discounted as a refractory colony, but our results show vector competence for ORL to be more nuanced and highly virus strain-dependent.

Our work highlights a potential vertical transmission system for DENV-4 persistence in the mosquito population in FL. We previously identified and fully sequenced a DENV-4 strain with high identity to our DENV-4 from Haiti in *Ae. aegypti* from Manatee Co. in 2016 and 2017, in the absence of a human case across both years [40]. It is possible that there were unreported asymptomatic dengue cases [57] that acted as seeding events in Manatee Co., but this cannot be known with certainty unless paired with serology studies. However, DENV-3 maintenance by vertical transmission in *Ae. albopictus* from Brazil occurred in a period without autochthonous transmission [58]. Additionally, experimental infection of field-acquired *Ae. aegypti* has shown efficient vertical and venereal transmission of DENV-2 during second and third egg-laying cycles, especially when virus dissemination was high [59]. Manatee Co. (MAN) had significantly higher DENV-1 H and DENV-4 H IRs compared to DENV-2 L, DENV-3 L, and DENV-4 L, but did not have significantly higher TPs than those same groups. The high midgut IR and infection intensity of DENV-4 H in MAN and an unremarkable TP or saliva infection intensity suggest that vertical transmission may have been favored to maintain this virus in nature. Similarly, our research group recently showed that an infectious clone of the DENV-4 sequence found in Manatee Co. *Ae. aegypti* is capable of being both horizontally and vertically transmitted [60], and we also found DENV-4 H to be vertically transmitted at a higher rate than DENV-4 L in ORL [17]. A current limitation of our work is that we were unable to obtain and test low passage strains of patient-derived DENV-2 and DENV-3. In the future, we aim to include low passage DENVs across all four serotypes to understand if the trends uncovered herein persist across all laboratory and field virus pairings.

We noted that the presence of both the 1016 and 1534 pyrethroid-resistant *kdr* SNPs was associated with the most refractory, lowest DENV susceptible phenotypes of the field lines. This trend is opposite to early reports showing insecticide resistance increases virus dissemination and competence [37,38]. Those laboratory studies differ from ours in that one study actively selected for phenotypic pyrethroid resistance in a field *Ae. aegypti* colony and then evaluated DENV-1 dissemination in mosquito bodies of F_13_ and F_20_ generations [37]. The other study backcrossed ORL to obtain both a pyrethroid-susceptible and a pyrethroid-resistant colony, in which they evaluated ZIVK competence [38]. Our study used lower generation field-derived mosquitoes (F_2_ and F_7_) and did not select for phenotypic pyrethroid resistance. Without a direct assessment of resistance phenotype, we cannot say definitively that possessing *kdr* alleles conferred insecticide resistance or that resistance is correlated with higher vector competence. However, it is still important to consider that in nature, mosquitoes from these collection sites are likely exposed to pyrethroids from vector control mediated by a combination of publicly funded control programs and private pesticide applicators. Considering, within mosquito population structuring has been observed previously [33], additional studies are needed to comparatively measure other components of vectorial capacity, such as differences in host-seeking behavior or locomotor activity, longevity, and vector competence across multiple mosquito populations between regions, e.g., St. Johns Co. vs. Miami-Dade Co.

We showed that DENV serotype-specific variation in vector competence across four field *Ae. aegypti* populations from FL. Vector competence was highest for the two south FL mosquito groups, from Miami-Dade and Collier Co’s, and for the low passage field strains isolated from human specimens from Haiti (DENV-1 H and DENV-4 H). These observations emphasize the importance of choosing geographically and medically relevant mosquito and virus strains in vector competence studies to estimate more accurately DENV risk. Moreover, these data offer practical information to pinpoint areas and human populations at increased DENV risk. We expect that allocating resources proactively towards highly targeted vector control efforts in FL that have *Ae. aegypti* with high DENV transmission potential can prevent future or stop dengue outbreaks in FL.

## Figures and Tables

**Table 1:** Pairwise comparisons of DENV infection rates and transmission potentials of Florida *Aedes aegypti* populations reveal virus-specific and mosquito population-dependent differences.

	Mosquito Strain/ DENV Strain *	ORL	SJ	MAN	COL	MD
Infection Rates (%)	DENV-1 H	^a^ 97 _A_	^acd^ 26 _B_	^ad^ 74 _AB_	^ad^ 35 _B_	^a^ 67 _AB_
	DENV-1 L	^b^ 10	0	0	^b^ 2	0
	DENV-2 L	^c^ 28	^c^ 24	^b^ 27	^a^ 39	^b^ 40
	DENV-3 L	^bc^ 20	^b^ 5	^bc^ 9	^d^ 10	^c^ 22
	DENV-4 H	^d^ 61 _A_	^d^ 46 _A_	^d^ 63 _A_	^c^ 64 _AB_	^d^ 86 _B_
	DENV-4 L	^bc^ 21 _AB_	^bc^ 7 _A_	^b^ 17 _AB_	^abd^ 21 _AB_	^bc^ 29 _B_
Transmission Potentials (%)	DENV-1 H	^a^ 57 _A_	8 _AB_	17 _B_	^ab^ 20 _AB_	^a^ 42 _A_
	DENV-1 L	^ab^ 6	0	0	^ab^ 2	0
	DENV-2 L	^b^ 4	5	10	^a^ 11	^bc^ 14
	DENV-3 L	^b^ 3	0	2	^a^ 2	^bc^ 5
	DENV-4 H	^b^ 12 _ABD_	6 _A_	27 _D_	^b^ 43 _C_	^ac^ 44 _BCD_
	DENV-4 L	^b^ 4 _AB_	0	4 _AB_	^ab^ 11 _B_	^b^ 2 _A_

^abc^ Lowercase alphabetical superscripts represent infection rate/transmission potential comparisons across virus strains for each column for the same mosquito strain. Values denoted with different letters (a, b, c, or d) have significantly different infection or transmission potentials. Columns without superscripts had no significant differences between any comparison group. ^ABC^ Uppercase alphabetical subscripts represent infection rate/transmission potential comparisons across mosquito strains for each row for the same virus strain. Values with different letters (A, B, C, or D) have significantly different infection or transmission potentials. Rows without subscripts had no significant differences between any comparison group. * ORL was the most pyrethroid-susceptible, MAN was the second most pyrethroid-susceptible, COL was moderately pyrethroid resistant, MD was the second most pyrethroid-resistant and SJ was the most pyrethroid-resistant, based on *kdr* genotype frequencies.

**Table 2:** Average DENV titer (log_10_ PFU/mL) across *Aedes aegypti* strains in Florida reveals higher titers for the laboratory colony (ORL) as well as increased midgut titers for low passage DENV-4.

	Mosquito Strain/ DENV Strain *	ORL	SJ	MAN	COL	MD
Midgut (average [SD])	DENV-1 H	^a^ 3.03 (0.65) _A_	2.13 (0.92) _B_	_a_ 2.18 (0.80) _B_	_a_ 2.57 (0.65) _AB_	^a^ 2.18 (0.60) _B_
	DENV-1 L	--	--	--	--	--
	DENV-2 L	^b^ 2.46 (1.41)	2.00 (1.16)	_ab_ 2.64 (1.29)	_a_ 2.41 (1.33)	^a^ 2.09 (1.14)
	DENV-3 L	^ac^ 3.51 (1.40) _A_	3.05 (0.49) _AB_	_ab_ 2.54 (0.68) _AB_	_ab_ 3.24 (0.62) _A_	^a^ 1.75 (0.74) _B_
	DENV-4 H	^c^ 3.43 (0.85) _A_	2.37 (0.71) _B_	_b_ 3.02 (0.97) _AC_	_b_ 3.28 (0.76) _A_	^b^ 2.73 (1.09) _BC_
	DENV-4 L	^ab^ 2.80 (0.56) _A_	1.60 (0.17) _B_	_a_ 2.25 (1.21) _AB_	_ab_ 2.67 (0.71) _AB_	^ab^ 2.36 (0.63) _AB_
Saliva (average [SD])	DENV-1 H	^a^ 1.48 (0.96)	0.65 (0.74)	^ab^ 0.95 (0.94)	0.99 (0.72)	^a^ 1.22 (0.84)
	DENV-1 L	^b^ 0.36 (0.41)	--	--	0.11	--
	DENV-2 L	^b^ 0.22 (0.11)	1.14 (1.66)	^ab^ 0.89 (0.48)	0.74 (0.60)	^b^ 0.19 (0.23)
	DENV-3 L	0.95	--	^ab^ 1.77	0.85	^ab^ 1.2 (0.83)
	DENV-4 H	^a^ 1.55 (0.55) _A_	0.51 (0.86) _AB_	^a^ 0.37 (0.72) _B_	0.85 (0.84) _B_	^ab^ 0.87 (0.87) _AB_
	DENV-4 L	^ab^ 1.56 (0.26)	--	^b^ 1.63 (1.96)	1.43 (0.84)	^ab^ 0.83

-- Signifies that titer measurements were not obtained for these groups due to the qualitative nature of readouts from cell culture. ^abc^ Lowercase alphabetical superscripts represent average titer comparisons across virus strains for each column for the same mosquito strain. Values with different letters (a, b, or c) have significantly different infection intensities. Columns without superscripts had no significant differences between any comparison group. ^ABC^ Uppercase alphabetical subscripts represent average titer comparisons across mosquito strains for each row for the same virus strain. Values with different letters (A, B, or C) have significantly different infection intensities. Rows without subscripts had no significant differences between any comparison group * ORL was the most pyrethroid-susceptible, MAN was the second most pyrethroid-susceptible, COL was moderately pyrethroid resistant, MD was the second most pyrethroid-resistant and SJ was the most pyrethroid-resistant, based on *kdr* genotype frequencies.

## Data Availability

Data can be made available upon request.

## Appendix A

**Table A1:** Dengue virus (DENV) strains and sources used in experiments.

Virus	Strain	Source	Year	GenBank ID	Average Blood Meal Titer (PFU/ mL)	Stock Titer (PFU/ mL)
DENV-1 H	Haiti/1207/2014	Field isolate	2014	KT279761.2	6 × 10^6^	8.71 × 10^6^
DENV-1 L	NR-82 Hawaii	ATCC	1944	KM204119 ^a^	6 × 10^5^	9.5 × 10^5^
DENV-2 L	NR-84 New Guinea C [NGC]	ATCC	1944	KM204118 ^a^	5 × 10^6^	6.17 × 10^6^
DENV-3 L	NR-80 Philippines/H87	ATCC	1956	KU050695 ^a^	6.3 × 10^6^	8.43 × 10^6^
DENV-4 H	Haiti/0075/2015	Field isolate	2015	MK514144.1	2.5 × 10^6^	1.68 × 10^7^
DENV-4 L	NR-86 H241 [TC]	ATCC	1956	KR011349 ^a^	1.6 × 10^6^	8.7 × 10^6^

^a^ Reference genomes for laboratory viruses [52].

**Table A2:** Primer and probe sequences for pan-DENV, CFAV, and *kdr* allele detection in *Aedes aegypti*.

Primer Name	Sequence	Citation
DENV Forward 1	5′-AGGACYAGAGGTTAGAGGAGA-3′	
DENV Reverse 1	5′-CGYTCTGTGCCTGGAWTGAT-3′	[61]
DENV Probe 1	5′-FAM-ACAGCATATTGACGCTGGGARAGACC-BHQ1-3′	
DENV Forward 2	5′ GGACTAGAGGTTAGAGGAGACCCC-3′	[62]
DENV Reverse 2	5′-GAGACAGCAGGATCTCTGGTC-3′	
DENV Probe 2	5′-FAM-AGCATATTGACGCTGGGA-BHQ1-3′	
CFAV E Forward	5′-GCTTCAAGTGGGGGATTGGA -3′	[22]
CFAV E Reverse	5′- CAACTTTCTCCATGCCGTGC -3′	
V1016F	5′- GCGGGCAGGGCGGCGGGGGCGGGGCCACAAATTGTTTCCCACCCGCACCGG -3′	
I1016F	5′-GCGGGCACAAATTGTTTCCCACCCGCACTGA-3′	[43]
1016R	5′-GGATGAACCSAAATTGGACAAAAGC -3′	
F1534F	5′ GCGGGCTCTACTTTGTGTTCTTCATCATATT -3′	
C1534F 1534R	5′-GCGGGCAGGGCGGCGGGGGCGGGGCCTCTACTTTGTGTTCTTCATCATGTG-3′ 5′-TCTGCTCGTTGAAGTTGTCGAT-3′	[44]
		

**Table A3:** Sample sizes per experimental group of *Aedes aegypti* exposed to dengue viruses.

Mosquito Strain/DENV Strain	ORL	SJ	MD	COL	MAN
DENV-1 H	46/44/26	43/12/4	46/31/19	29/8/5	32/21/6
DENV-1 L	66/6/3	46/0/0	36/0/0	29/1/1	31/0/0
DENV-2 L	50/15/2	46/11/2	53/21/6	43/19/6	35/10/4
DENV-3 L	52/8/1	40/2/0	49/12/3	47/5/1	44/4/1
DENV-4 H	55/33/7	35/17/2	53/46/24	40/28/21	39/25/11
DENV-4 L	51/11/2	37/3/0	54/16/1	36/8/4	48/8/2

Order of numbers reflects the number of DENV-exposed *Aedes aegypti*/number of mosquitoes with DENV in their midgut 14 days post-exposure/number of mosquitoes with DENV in their saliva 14 days post-exposure. Sample sizes reflect the total number of mosquitoes in each category across three experimental replicates.

**Table A4:** Knockdown resistance (*kdr)* genotype percentages for Florida *Aedes aegypti* field populations (St. Johns, Manatee, Miami-Dade, Collier) and a laboratory colony (Orlando; ORL).

Genotypes	St. Johns	Manatee	Miami-Dade	Collier	ORL
VVFF	0	0	0	0	100
VVFC	0	0	7	3	0
VVCC	0	37	10	6	0
VIFF	0	0	0	0	0
VIFC	0	0	7	19	0
VICC	0	50	38	52	0
IIFF	0	0	0	0	0
IIFC	0	0	0	3	0
IICC	100	13	38	16	0
